# Moderately high folate level may offset the effects of aberrant DNA methylation of P16 and P53 genes in esophageal squamous cell carcinoma and precancerous lesions

**DOI:** 10.1186/s12263-020-00677-x

**Published:** 2020-09-29

**Authors:** Shaokang Wang, Da Pan, Ming Su, Guiling Huang, Guiju Sun

**Affiliations:** 1 Key Laboratory of Environmental Medicine and Engineering of Ministry of Education, and Department of Nutrition and Food Hygiene, School of Public Health, Southeast University, Nanjing, 210009 P. R. China; 2 Huai’an District Center for Disease Control and Prevention, Huai’an, 223200 P. R. China; 3 Jiangsu Research Center for Primary Health Development and General Practice Education, Jiangsu Vocational College of Medicine, Yancheng, 224005 P. R. China

**Keywords:** Esophageal squamous cell carcinoma, Esophageal precancerous lesion, Serum folate, Dietary intake, DNA methylation, Gene-nutrition interaction

## Abstract

**Background:**

This study evaluated gene-nutrition interactions between folate and the aberrant DNA methylation of tumor suppressor genes in different stages of carcinogenesis of esophageal squamous cell carcinoma (ESCC).

**Methods:**

Two hundred ESCC cases, 200 esophageal precancerous lesion (EPL) cases, and 200 controls matched by age (± 2 years) and gender were used for this study. Baseline data and dietary intake information was collected via questionnaire. The serum folate levels and methylation status of promoter regions of p16 and p53 were detected.

**Results:**

The interactions of increased serum folate level with unmethylated p16 and p53 promoter regions were significantly associated with a reduced risk of both EPL and ESCC (*p* for interaction < 0.05). The interactions of the lowest quartile of serum folate level with p16 or p53 methylation was significantly associated with an increased risk of ESCC (OR = 2.96, 95% CI, 1.45–6.05; OR = 2.34, 95% CI, 1.15–4.75). An increased serum folate level was also related to a decreasing trend of EPL and ESCC risks when p16 or p53 methylation occurred. The interaction of spinach, Chinese cabbage, liver and bean intake with unmethylated p16 and p53 was significantly associated with a reduced risk of EPL or ESCC (*p* for interaction < 0.05).

**Conclusions:**

The interactions between a high folate level and unmethylated p16 and p53 promoter regions may have a strong preventive effect on esophageal carcinogenesis. Additionally, a high folate level may offset the tumor-promoting effects of aberrant DNA methylation of the genes, but it is also noteworthy that a very high level of folate may not have a protective effect on EPL in some cases.

## Introduction

According to global cancer statistics in 2018, esophageal cancer (EC) was the seventh most common type of cancer worldwide. It is aggressive in nature and is often diagnosed in late stages, rendering it the sixth leading cause of cancer death worldwide with a low survival rate [1]. In developing countries such as China, esophageal squamous cell carcinoma (ESCC) accounts for approximately 90% of new EC cases each year, which renders it the most prevalent histological sub-type of EC in these countries [2]. The best results of reducing the proportion of cancer patients who are diagnosed at late stages are associated with an early diagnosis, which is commonly known as the “early stages” [3]. Therefore, esophageal squamous dysplasia, which is the recognized esophageal precancerous lesion (EPL) for ESCC, has been of greater interest over the last decade [4–10]. A previous study indicated that 24% of mild dysplasia, 50% of moderate dysplasia, and 74% of severe dysplasia could develop ESCC during a 3.5-year period [6]. It is commonly known that although the dominant factors vary among different regions, ESCC is caused by a complex combination of many genetic, environmental and dietary factors, and EPL may share similar protective or risk factors with ESCC [2, 11]. However, it should be pointed out that the possibility of a preventive or harmful effect of some micronutrients (especially some B vitamins) on carcinogenesis within the extremely long latency period time of different cancer development stages (i.e., before or after the establishment of precancerous lesions) cannot be ignored [12].

B vitamins which are involved in the one-carbon metabolism pathway have been demonstrated to play key roles in DNA synthesis, repair and methylation [13]. Folate is an essential methyl donor in the folate cycle; thus, folate deficiency can lead to the aberrant expression of critical proto-oncogenes and tumor suppressor genes by regulating DNA methylation leading to an increased risk of cancer [14]. However, several epidemiological studies reported that factors contributing to folate levels provide no protection against some cancers, suggesting that folate may even stimulate cancer progression [15–20]. As folate is also essential for DNA synthesis, repair and methylation in dysplasia cells and cancer cells, it has been hypothesized that folate may play a dual role in cancer development: preventing tumor initiation in the early carcinogenesis, yet promoting carcinogenesis after the establishment of precancerous lesions, and the “double-edged sword” effect may be time- and dose dependent [21–28]. However, the available data remains incomplete and the results are limited, thus it is necessary to conduct a study to investigate the roles of folate in the different stages of ESCC, in order to assess whether there is a preventive effect on initial development of EPL rather than progression of ESCC.

In addition, carcinogenesis is associated with a complex combination of genetic, environmental, and dietary factors; thus, these factors should be considered equally to achieve better health outcomes for populations. Previous studies were mostly directed at either the effects of dietary and environmental factors alone or at specific genes only rather than all factors together. In the last decade, gene-nutrition interactions have been studied. For example, our recent study indicated that the serum levels of vitamin B_2_ and B_12_ and genetic polymorphism may interact in ways which has a different effect on the risks of EPL and ESCC [9]. Previous molecular epidemiological studies also investigated the gene-nutrition interactions between dietary folate intake and aberrant DNA methylation in ESCC based on a food frequency questionnaire (FFQ) [29, 30], but the exact serum level of folate was never assessed in these interactions. Therefore, folate may influence the development of ESCC by directly affecting the expression of genes in critical metabolic pathways such as the folate cycle. Scientific research on ESCC necessitates better understanding of the folate-dependent interactions based not only dietary intake but also on the serum concentration of folate at the genetic, epigenetic and molecular levels.

Gene p16 (cyclin-dependent kinase inhibitor 2A) and p53 (tumor protein p53) are important tumor suppressor genes located on chromosome 9p21 and 17p13, respectively. Aberrant DNA hypermethylation of promoter regions in the tumor suppressor genes p16 and p53 was reported to be responsible for the silence and inactivation of the corresponding gene which is involved in carcinogenesis of esophagus [9, 31–35], suggesting that epigenetic alteration in p16 and p53 is involved in the pathogenesis of ESCC. Additionally, a previous study also suggested that aberrant DNA hypermethylation may be a consequence of various environmental and dietary factors dependent on the specific region resulting in an elevated susceptibility to ESCC [31]. Therefore, the possible association of epigenetic events along with other environmental/dietary/nutritional factors should not be ruled out.

In the present study, we have examined the serum level of folate, promoter methylation status of frequently methylated tumor suppressor genes p16 and p53 using blood samples, and investigated the intake of foods rich in folate via a validated qualitative FFQ among healthy controls, EPL cases, and ESCC cases. This allowed us to explore the gene-nutrition interaction between folate and aberrant DNA methylation in the different stages of carcinogenesis of ESCC.

## Materials and methods

### Study subjects and EPL diagnosis

Huai’an District, an inland rural area located in the Northern Jiangsu Province of China (Supplementary Figure S1), is a high risk area for ESCC, with crude incidence and mortality rates of 96.15/100,000 and 63.25/100,000, respectively [8]. Previous studies reported a distinct epidemiological pattern of EPL in Huai’an District and the consumption of alcohol and tobacco products, which has been the key risk factors in many regions. However, these factors only played a minor role in the EPL risk, with other factors such as genetic polymorphism, environmental exposures, and dietary factors reported to take the main responsibility for esophageal carcinogenesis in this endemic region [7–10, 36].

Considering the importance of promoting the prevention strategies, definitive treatment of ESCC, and the cost-effectiveness, the government and Cancer Foundation of China established the Early Diagnosis and Early Treatment Project of Esophageal Cancer (EDETPEC) in the Huai’an District from 2010. Baseline data and blood samples were collected immediately when participants were recruited. All the participants in the EDETPEC received a routine endoscopy examination. Esophageal mucosa was stained with 10 ml of 1.2% Lugol’s iodine solution for observation; then, the normal esophageal mucosa (which is iodine-positive) would turn brown, while the iodine-negative dysplastic lesions would remain unstained and the tissues would be sampled. Subsequently, biopsies were oriented on filter paper, placed in 10% phosphate-buffered formalin, sent to the pathology laboratory, and then processed to paraffin blocks, prepared on the slides, and stained with hematoxylin-eosin for histopathological diagnosis. EPL can be classified into mild, moderate, and severe dysplasia referring to the histological criteria of dysplasia, which requires the loss of normal cell polarity, the presence of nuclear atypia, and abnormal tissue maturation without invasion of epithelial cells through the basement membrane [11].

Two hundred diagnosed EPL cases and 200 healthy controls aged between 40 and 79 were selected from approximately 5000 participants who had been recruited in the EDETPEC from January 2010 to December 2013. During the same period of time, 200 newly diagnosed ESCC cases were selected from Huai’an District cancer registry system, and the groups of 200 EPL cases, 200 ESCC cases, and 200 normal controls were matched by age (± 2 years) and gender. All the enrolled subjects were required not to have a history of cancer, take B vitamins supplements recently and receive esophageal cancer surgery, radiotherapy or chemotherapy. The study protocol was approved by the Institutional Review Board of Southeast University Zhongda Hospital with approval number 2012ZDllKY19.0, in accordance with the Declaration of Helsinki. All the subjects signed written informed consent.

### Data and sample collection

After written informed consent was obtained, trained interviewers collected epidemiological data including socio-demographics, lifestyle, and eating habits by face-to-face interviews using a questionnaire. Subjects who smoked at least one cigarette or consumed at least one alcoholic beverage per day (on average) continuously for at least half a year were defined as “smokers” and “drinkers” respectively. As described previously [8], dietary intake was estimated by using a validated qualitative FFQ, which covered 12 specific food types and 45 specific food items that are commonly consumed in this region. The food intake frequencies were as follows: “never,” “less than once per week,” and “once per week or more.” According to China Food Composition [37, 38], the folate content in 31 specific food items listed in the FFQ are shown in Supplementary Table S1. The foods that are rich in folate will be the items considered during the analyses. In addition, blood samples were taken from subjects who had undergone 12 h of fasting and centrifuged at 4000 rpm for 5 min to obtain samples of separated serum and leukocyte. The samples were then refrigerated at − 80 °C.

### Determination of serum folate level

A double-antibody-sandwich enzyme-linked immunosorbent assay (ELISA) was used to determine the serum level of folate. Folate (human) ELISA kits (Shanghai FanKe industrial Co., Ltd. Shanghai, China) were used in the procedure. All operations were in strict accordance with manufacturer's instructions. Optical density (OD) values at a wavelength of 450 nm were measured using a multimode microplate reader (Mithras LB 940, Berthold Technologies, Bad Wildbad, Germany), and then the serum level of folate was calculated accordingly.

### Detection of DNA methylation

Genomic DNA was extracted from leukocyte samples by using Wizard® Genomic DNA Purification kit (A1120, Promega, WI, USA). The methylation status at the promoter region of p16 and p53 was detected by methylation-specific polymerase chain reaction (MSP) after sodium bisulfate modification of DNA [39]. MSP condition was initial denaturation at 95 °C for 5 min, followed by 35 cycles of denaturation at 95 °C for 30 s, annealing at 50 °C for 30 s, extension at 72 °C for 60 s, and final extension at 72 °C for 10 min. Methylation-specific primers for p16 and p53 are shown in Table 1. MSP products electrophoresed on 2% agarose gel and visualized under UV illumination after being stained with ethidium bromide [9].

**Table 1:** Primers used for PCR

Genes^a^	S/AS^b^	Primer sequences	Product size (bp)
p16M	S	5′-TTA TTA GAG GGT GGG GCG GAT CGC-3′	150
	AS	5′-GAC CCC GAA CCG CGA CCG TAA-3′	
p16U	S	5′-TTA TTA GAG GGT GGG GTG GAT TGT-3′	151
	AS	5′-CAA CCC CAA ACC ACA ACC ATA A-3′	
p53M	S	5′-GTA GTT TGA ACG TTT TTA TTT TGG C-3′	115
	AS	5′-CCT ACT ACG CCC TCT ACA AAC G-3′	
p53U	S	5′-GTA GTT TGA ATG TTT TTA TTT TGG T-3′	115
	AS	5′-CCT ACT ACA CCC TCT ACA AAC A-3′	

^a^*M* methylated, *U* unmethylated

^b^*S/AS* sense/antisense

### Statistical analysis

Data collected via questionnaire were double-entered and validated in an established database using Epidata version 3.1 (EpiData Association, Odense, Denmark). SPSS version 17.0 (SPSS, Chicago, IL, USA) was used to perform statistical analyses. The differences in the serum folate level, distribution of socio-demographic characteristics, and methylation status among healthy controls were analyzed for EPL cases and ESCC cases using Kruskal-Wallis test, Mann-Whitney *U* test, Chi-square (*χ*^2^) test and one-way analysis of variance (ANOVA), wherever appropriate. The continuous variables of serum folate level were categorized into quartiles (Q1, Q2, Q3, and Q4) according to the folate levels of healthy control group. Binary logistic regression was conducted to assess the association between serum folate level and risk of EPL or ESCC. Gene-nutrition interaction between folate and aberrant DNA methylation in EPL and ESCC was investigated using a multiplicative interaction model based on logistic regression. Analyses were performed with adjustment for confounding variables including gender, age, tobacco smoking, and alcohol drinking. Results were expressed by calculated odds ratio (OR) and its corresponding 95% confidence interval (CI). The associations between dietary intake of each food item and the risks of EPL and ESCC were evaluated without adjustment for confounders using the univariate logistic regression model to obtain crude OR at the beginning. Then, the food items which showed statistical significance were selected for further multiple logistic regression analysis with adjustment for confounding variables. Subsequently, the interactions between dietary factors which had statistically significant adjusted ORs and aberrant DNA methylation in EPL and ESCC were evaluated using the multiplicative interaction model. Statistical significance was considered as *p* < 0.05 (two-tailed).

## Results

### Socio-demographic characteristics of the subjects

In this study, 200 healthy controls, 200 diagnosed EPL cases, and 200 newly diagnosed ESCC cases matched by age (± 2 years) and gender were involved, 106 pairs of which were males and 94 pairs were females. The average ages of the three groups were 62.89 ± 5.10, 61.26 ± 6.82, and 62.57 ± 6.65 years in male; 62.46 ± 5.68, 61.31 ± 6.38, and 61.94 ± 6.34 years in female; and 62.69 ± 5.37, 61.29 ± 6.60, and 62.28 ± 6.49 years in total. There was no statistical significant difference in age among three groups (*p* > 0.05, ANOVA).

### Serum folate level and DNA methylation status of the subjects

The serum levels of folate and DNA methylation status of p16 and p53 in normal controls, EPL cases and ESCC cases are shown in Table 2. The Kruskal-Wallis test showed that there was a statistically significant difference in serum folate level among the three groups (*p* < 0.001). The Mann-Whitney *U* test showed that the serum folate level of healthy controls was significantly higher than that of both EPL and ESCC cases (*p* < 0.05). Hypermethylation of p16 and p53 promoter regions occurred in 35.0% and 36.5% of healthy controls, 46.0% and 42.0% of EPL cases, and 63.0% and 56.5% of ESCC cases, respectively. The Chi-square (*χ*^2^) test illustrated that there were statistically significant differences in p16 and p53 promoter methylation among the three groups (*χ*^2^ = 31.89, *p* < 0.05; *χ*^2^ = 16.57, *p* < 0.05). Additionally, p16 methylation was more frequently detected in both EPL and ESCC cases when compared with healthy controls (*χ*^2^ = 5.02, *p* < 0.05; *χ*^2^ = 31.37, *p* < 0.001). Meanwhile, p53 methylation was more frequently detected in ESCC cases when compared with healthy controls (*χ*^2^ = 15.28, *p* < 0.001), whereas no significant difference was found between healthy controls and EPL cases (*χ*^2^ = 1.05, *p* > 0.05).

**Table 2:** Serum folate level (median (25th–75th) and DNA methylation status (*n* (%)) in the three groups

Variables	Control (*n* = 200)	EPL (*n* = 200)	ESCC (*n* = 200)	*p* value
Serum folate (μg/L)	29.14 (24.43–36.24)	25.72 (21.29–29.47)^a^	24.79 (20.73–29.85)^a^	< 0.001^*^
No. of p16 methylation	70 (35.0%)	92 (46.0%)^b^	126 (63.0%)^b^	< 0.05^†^
No. of p53 methylation	74 (36.5%)	84 (42.0%)	113 (56.5%)^b^	< 0.05^†^

^*^p value of Kruskal-Wallis test

^†^*p* value of Chi-square (*χ*^2^) test

^a^Compared with control group, *p* < 0.05 (Mann-Whitney *U* test)

^b^Compared with control group, *p* < 0.05 (Chi-square (*χ*^2^) test)

### The association between serum folate level and risk of EPL and ESCC

As shown in Table 3, serum folate concentration was classified into quartiles based on the folate level of healthy controls. Compared with the lowest quartile, the results indicated that both the third and highest quartiles of serum folate were inversely associated with the risk of both EPL (OR = 0.32, 95% CI 0.16–0.62; OR = 0.26, 95% CI 0.13–0.54) and ESCC (OR = 0.51, 95% CI 0.29–0.89; OR = 0.15, 95% CI 0.07–0.32) with adjustment for gender, age, tobacco smoking, and alcohol drinking.

**Table 3:** Associations between serum folate level and risk of EPL and ESCC

Serum folate (μg/L)	Control	EPL			ESCC		
	*n* (%)	*n* (%)	OR (95% CI)^a^	*p* value	*n* (%)	OR (95% CI)^a^	*p* value
Q1 (< 24.43)	50 (25.0)	80 (40.0)	1.00 (referent)		90 (45.0)	1.00 (referent)	
Q2 (24.43–29.14)	50 (25.0)	68 (34.0)	0.73 (0.41–1.30)	0.290	55 (27.5)	0.67 (0.39–1.152)	0.147
Q3 (29.14–36.24)	50 (25.0)	24 (12.0)	0.32 (0.16–0.62)	0.001	44 (22.0)	0.51 (0.29–0.89)	0.017
Q4 (> 36.24)	50 (25.0)	28 (14.0)	0.26 (0.13–0.54)	< 0.001	11 (5.5)	0.15 (0.07–0.32)	< 0.001

^a^Adjusted for gender, age, tobacco smoking, and alcohol drinking

### Gene-nutrition interaction between serum folate level and DNA methylation

The gene-nutrition interaction between folate and DNA methylation in EPL and ESCC was assessed using a multiplicative interaction model based on logistic regression. As shown in Tables 4 and 5, the interactions of an increased serum folate level with unmethylated p16 and p53 promoter regions was significantly associated with a reduced risk of both EPL and ESCC (*p* for interaction < 0.05). The interactions of the lowest quartile of serum folate level with p16 or p53 methylation were significantly associated with increased risk of ESCC (OR = 2.96, 95% CI 1.45–6.05; OR = 2.34, 95% CI 1.15–4.75). However, the results of adjusted ORs suggested that an increased serum folate level was related to a decreasing trend of the risk for both EPL and ESCC when p16 or p53 methylation occurred. In addition, the third quartile of serum folate level was significantly associated with reduced risk of EPL when p16 or p53 methylation occurred (OR = 0.27, 95% CI 0.10–0.72; OR = 0.23, 95% CI 0.09–0.61). The highest quartile of serum folate level was significantly associated with a reduced risk of ESCC when p53 methylation occurred (OR = 0.25, 95% CI 0.08–0.74). Interestingly, in the case of hypermethylation of p16 and p53 promoter regions, there was no association between the highest quartile of serum folate level and EPL risk.

**Table 4:** Association between serum folate level with p16 methylation status and the risk of EPL and ESCC

Serum folate (μg/L)	EPL [OR (95% CI)^a^, *p* for interaction]		ESCC [OR (95% CI)^a^, *p* for interaction]	
	p16U	p16M	p16U	p16M
Q1 (< 24.43)	1.00 (reference)	1.63 (0.79–3.35), 0.183	1.00 (reference)	2.96 (1.45–6.05), 0.003
Q2 (24.43–29.14)	0.82 (0.42–1.59), 0.559	1.60 (0.75–3.42), 0.226	0.57 (0.27–1.20), 0.137	2.12 (0.98–4.58), 0.056
Q3 (29.14–36.24)	0.41 (0.19–0.87), 0.021	0.27 (0.10–0.72), 0.009	0.52 (0.24–1.11), 0.090	1.31 (0.61–2.80), 0.489
Q4 (> 36.24)	0.35 (0.16–0.76), 0.008	0.62 (0.25–1.51), 0.294	0.15 (0.05–0.43), < 0.001	0.39 (0.13–1.13), 0.082

*U* unmethylated, *M* methylated

^a^Adjusted for gender, age, tobacco smoking, and alcohol drinking

**Table 5:** Association between serum folate level with p53 methylation status and the risk of EPL and ESCC

Serum folate (μg/L)	EPL [OR (95% CI)^a^, *p* for Interaction]		ESCC [OR (95% CI)^a^, *p* for Interaction]	
	p53U	p53M	p53U	p53M
Q1 (< 24.43)	1.00 (reference)	1.27 (0.62–2.61), 0.518	1.00 (reference)	2.34 (1.15–4.75), 0.019
Q2 (24.43–29.14)	0.77 (0.40–1.49), 0.440	1.30 (0.62–2.73), 0.494	0.66 (0.33–1.33), 0.246	1.43 (0.67–3.07), 0.361
Q3 (29.14–36.24)	0.38 (0.18–0.80), 0.011	0.23 (0.09–0.61), 0.003	0.49 (0.23–1.03), 0.060	1.04 (0.49–2.19), 0.923
Q4 (> 36.24)	0.38 (0.18–0.80), 0.011	0.41 (0.16–1.00), 0.050	0.16 (0.06–0.44), < 0.001	0.25 (0.08–0.74), 0.013

*U* unmethylated, *M* methylated

^a^Adjusted for gender, age, tobacco smoking, and alcohol drinking

### Interaction between dietary factors and DNA methylation

The associations between dietary intake of each food item and the risks of EPL and ESCC were analyzed without adjustment for confounding variables using the univariate logistic regression model at the beginning, and the obtained crude ORs with statistical significance are shown in Supplementary Table S2. Then, the food items which showed statistical significance in the univariate logistic regression model were selected for further multiple logistic regression analysis with adjustment for confounding variables. The obtained significant adjusted ORs are shown in Supplementary Table S3. Chinese cabbage, spinach, livers and beans, which all contain relatively high levels of folate (Supplementary Table S1), were found to be significantly associated with a reduced risk of EPL or ESCC (*p* < 0.05), and then selected for interaction analysis.

Briefly, Tables 6 and 7 illustrate that the interactions of spinach, Chinese cabbage, liver and bean intake with unmethylated p16 and p53 promoter regions were significantly associated with a reduced risk of EPL or ESCC (*p* for interaction < 0.05). The intake of Chinese cabbage was significantly associated with the reduced risk of EPL when p53 methylation occurred (OR = 0.29, 95% CI 0.11–0.78). The interactions of not consuming any Chinese cabbage or livers with p16 or p53 methylation was significantly associated with an increased risk of EPL or ESCC (*p* for interaction < 0.05), whereas the association between p16 or p53 methylation and EPL or ESCC risk was not statistically significant for those that consumed Chinese cabbage or livers once per week or more (*p* for interaction > 0.05). However, regardless of the intake frequency of beans, p16 methylation was significantly associated with an increased risk of ESCC (OR = 2.58, 95% CI 1.26–5.32; OR = 3.79, 95% CI 1.45–9.87; OR = 3.59, 95% CI 1.65–7.82).

**Table 6:** Association between foods rich in folate with p16 methylation status and the risk of EPL and ESCC

Food intake frequency	EPL [OR (95% CI)^a^, *p* for interaction]				ESCC [OR (95% CI)^a^, *p* for interaction]			
	p16U	*p* value	p16M	*p* value	p16U	*p* value	p16M	*p* value
Spinach								
Never	1.00 (reference)	-	0.80 (0.16–4.12)	0.790	1.00 (reference)	-	2.80 (0.78–10.02)	0.113
Less than once per week	1.20 (0.41–3.53)	0.740	1.52 (0.50–4.63)	0.459	0.34 (0.13–0.93)	0.035	0.76 (0.28–2.07)	0.592
Once per week or more	0.84 (0.29–2.41)	0.749	1.23 (0.41–3.64)	0.714	0.29 (0.11–0.75)	0.011	1.02 (0.39–2.63)	0.974
Chinese cabbage								
Never	1.00 (reference)	-	1.45 (0.92–2.29)	0.110	1.00 (reference)	-	3.27 (2.01–5.30)	< 0.001
Less than once per week	0.78 (0.25–2.42)	0.240	0.91 (0.22–3.77)	0.901	2.62 (0.96–7.19)	0.062	5.42 (1.68–17.51)	0.005
Once per week or more	0.22 (0.10–0.47)	<0.001	0.43 (0.17–1.10)	0.077	0.48 (0.23–1.03)	0.059	1.44 (0.64–3.28)	0.379
Livers								
Never	1.00 (reference)	-	1.58 (1.03–2.43)	0.038	1.00 (reference)	-	3.12 (1.99–4.87)	< 0.001
Less than once per week	0.36 (0.15–0.83)	0.017	0.71 (0.12–4.34)	0.710	0.82 (0.38–1.76)	0.609	6.01 (1.61–22.41)	0.008
Once per week or more	1.06 (0.30–3.79)	0.924	0.27 (0.03–2.42)	0.240	0.33 (0.04–2.87)	0.314	0.41 (0.05–3.75)	0.430
Beans								
Never	1.00 (reference)	-	0.76 (0.38–1.53)	0.445	1.00 (reference)	-	2.58 (1.26–5.32)	0.010
Less than once per week	1.14 (0.80–2.24)	0.712	1.99 (0.80–4.95)	0.137	1.22 (0.55–2.69)	0.626	3.79 (1.45–9.87)	0.006
Once per week or more	0.47 (0.24–0.93)	0.029	1.25 (0.59–2.63)	0.566	0.83 (0.39–1.75)	0.619	3.59 (1.65–7.82)	0.001

*U* unmethylated, *M* methylated

^a^Adjusted for gender, age, tobacco smoking, and alcohol drinking

**Table 7:** Association between foods rich in folate with p53 methylation status and the risk of EPL and ESCC

Food intake frequency	EPL [OR (95% CI)^a^, *p* for interaction]				ESCC [OR (95% CI)^a^, *p* for interaction]			
	p53U	*p* value	p53M	*p* value	p53U	*p* value	p53M	*p* value
Spinach								
Never	1.00 (reference)	-	0.39 (0.07–2.13)	0.277	1.00 (reference)	-	1.54 (0.44–5.32)	0.499
Less than once per week	1.04 (0.36–3.08)	0.937	1.01 (0.33–3.13)	0.980	0.28 (0.10–0.77)	0.014	0.51 (0.19–1.42)	0.199
Once per week or more	0.71 (0.24–2.04)	0.520	0.85 (0.28–2.52)	0.765	0.27 (0.11–0.71)	0.008	0.61 (0.23–1.59)	0.311
Chinese cabbage								
Never	1.00 (reference)	-	1.16 (0.73–1.83)	0.527	1.00 (reference)	-	2.19 (1.36–3.51)	0.001
Less than once per week	0.71 (0.23–2.20)	0.544	0.83 (0.20–3.42)	0.796	2.26 (0.84–6.07)	0.106	3.95 (1.22–12.84)	0.022
Once per week or more	0.23 (0.11–0.49)	< 0.001	0.29 (0.11–0.78)	0.014	0.40 (0.19–0.85)	0.017	1.01 (0.46–2.23)	0.985
Livers								
Never	1.00 (reference)	-	1.19 (0.78–1.83)	0.426	1.00 (reference)	-	2.10 (1.36–3.24)	0.001
Less than once per week	0.31 (0.13–0.73)	0.007	0.63 (0.10–3.84)	0.614	0.71 (0.34–1.49)	0.369	4.38 (1.16–16.50)	0.029
Once per week or more	0.94 (0.26–3.35)	0.925	0.24 (0.03–2.14)	0.199	0.26 (0.03–2.30)	0.227	0.33 (0.04–3.00)	0.324
Beans								
Never	1.00 (reference)	-	0.63 (0.31–1.27)	0.193	1.00 (reference)	-	1.44 (0.72–2.89)	0.301
Less than once per week	1.06 (0.54–2.10)	0.862	1.74 (0.69–4.34)	0.239	0.88 (0.41–1.89)	0.747	2.51(0.98–6.42)	0.056
Once per week or more	0.51 (0.26–0.99)	0.048	0.94 (0.44–2.03)	0.873	0.67 (0.33–1.35)	0.263	2.26(1.06–4.82)	0.462

*U* unmethylated, *M* methylated

^a^Adjusted for gender, age, tobacco smoking, and alcohol drinking

## Discussion

In this study, we attempted to investigate the gene-nutrition interaction with folate and aberrant DNA methylation in the different stages of carcinogenesis of ESCC by detecting serum folate level, promoter methylation status of frequently methylated tumor suppressor genes p16 and p53, and evaluating the intake frequency of foods rich in folate among healthy controls, EPL cases, and ESCC cases. The results indicated that methylation of p16 and p53 promoter regions were significantly associated with the risk of EPL or ESCC. A high serum folate level, high intake of foods rich in folate, and unmethylated p16 and p53 promoter regions may interact in ways that have a strong preventive effect on esophageal carcinogenesis. In addition, a high serum folate level and high intake of foods rich in folate may offset the tumor-promoting effects of aberrant DNA methylation of p16 and p53 genes.

To the best of our knowledge, the inactivation of critical tumor suppressor genes may contribute to cancer development. The tumor suppressor gene p16 inhibits cyclin D-dependent protein kinases, which plays a key role in cell cycle regulation by decelerating the G1-S transition [32]. The inactivation of p16 is associated with p16 promoter methylation and may be a frequent event in the endemic region. The tumor suppressor gene p53 regulates cell division by inhibiting the overly rapid or uncontrolled cell growth and proliferation [31]. Similarly, p53 promoter methylation results in deactivation and causes an increased risk of cancers such as breast carcinomas, gliomas, acute lymphoblastic leukemia, hepatocellular carcinomas, and ovarian cancer [40–44], as well as ESCC in the study area specifically. In addition, previous studies and meta-analysis reported that p53 codon 72 polymorphism is associated with ESCC risk, especially in Asian population [45–47]. A recent study demonstrated that compared with the p53 codon 72 Arg/Arg and Arg/Pro genotype, the Pro/Pro genotype was found to confer a significant risk toward ESCC development [31]. Thus, it is necessary to carry out further studies for evaluate the possible overlap and crosstalk between aberrant DNA methylation, genetic polymorphism and environmental/dietary/nutritional factors.

It has been demonstrated that epigenetic silencing of tumor suppressor genes caused by promoter hypermethylation is an early major event during carcinogenesis [30, 31], but the gene-nutrition interaction between folate and aberrant DNA methylation cannot be ruled out because folate is the primary methyl donor in the folate cycle which regulates DNA methylation of important proto-oncogenes and tumor suppressor genes [14]. DNA methylation has been found to occur at CpG dinucleotides by transferring a methyl group to the fifth position of a cytosine base by DNA methyltransferases [48]. Folate plays a critical role in the synthesis of S-adenosylmethonine (SAM) and the further conversion of S-adenosylhomocysteine (SAH) by providing a methyl group to DNA methylation and histones [49]. Therefore, folate deficiency may contribute to a perturbed expression of genes that are controlled by DNA methylation. For example, the expression levels of tumor suppressor genes, including p53 and p16, were reported to be reduced at the transcriptional level because of promoter hypermethylation as a result of folate deficiency. Conversely, the repletion with physiological folate and folate supplementation may downregulate proto-oncogenes and upregulate tumor suppressor genes due to the corresponding regulation of promoter methylation [50]. In addition, folate is the substrate for conversion of deoxyuridine monophosphage into deoxythymidine monophosphate, which is involved in thymine synthesis, as well as the formation and stability of DNA, RNA, and nucleoside triphosphates [51, 52]. Therefore, the deficiency of folate may also result in the incorporation of uracil instead of thymine into the DNA, which cause an altered DNA repair mechanism, chromosomal breaks, and mutations [53]. In other words, the intake and serum level of folate not only affects the folate metabolic pathway, but also influences the corresponding genetic and epigenetic pathways which are associated with malignant transformation and tumor progression [54]. This suggests that nutrition and genetics/epigenetics may have an even greater role to play in carcinogenesis than the single effect seen to date. Furthermore, 5,10-methylenetetrahydrofolate reductase (MTHFR) is the central enzyme in folate metabolism and is responsible for the circulation form of folate, as well as its activity in the DNA methylation process [9]. It has been reported that MTHFR C677T polymorphism and folate status can interact in ways which impact DNA methylation status as well, which in turn affects cancer risk [29, 55]. Furthermore, age is positively associated with an increased risk of cancer, with the mean age of the population studied being over 60 years old. Folate deficiency in an aged population results in aberrations in the methylation patterns, whereas the perturbed methylation pattern associated with older age has shown a role in promoting cancers [56, 57]. A previous study induced folate deficiency in weanling, young and adult male Wistar rat groups, and the results indicated that folate deficiency with older age commences toward the upregulation of proto-oncogenes (e.g., DNMT3a, DNMT3b, DNMT1 and MBD) and downregulation of tumor suppressor genes (e.g., p53, p16, and p15) at the transcriptional level. This interaction occurs by regulating promoter methylation, which suggests that the combined effect of folate deficiency and age on epigenetic alteration may contribute to the pathogenesis at cellular level [50].

Interestingly, as shown in Tables 4 and 5, the third quartile of serum folate level was significantly associated with a reduced risk of EPL, whereas in the case of hypermethylation of p16 or p53 promoter regions, no association between the highest quartile of serum folate level and EPL risk was observed. This suggests that a very high level of folate status may not have protective effect on EPL in some cases. As mentioned previously, the “double-edged sword” effect of folate on cancer development may be time and dose dependent [21–28], but there is still controversy in the literature over the possibility of both positive and potential negative effects of folate, especially folate supplementation [58]. A previous study which investigated an elderly Chilean subject group and indicated that a long-term high consumption of foods fortified with folate could increase circulating folate in serum and thus result in higher levels of gene-specific methylation. Conversely, the increased level of methylation in tumor suppressor genes (including p16) may inhibit the gene expression that are in turn associated with an elevated cancer risk [59]. In this study, although the individuals who took B vitamin supplements were not included, the relationship between serum folate status and cancer may still be one of the most controversial subjects in the field. The potential mechanism by which the effect of folate on cancer can be time and dose dependent has been suggested: folate intake is likely to play a protective role on carcinogenesis in normal cells. However, it might also contribute to the progression of neoplastic lesions such as EPL because an abundant supply of folate is required in rapidly dividing cells for supporting de novo nucleotide biosynthesis and DNA methylation, which are critical for cell division [58]. The above mechanism also serves as the basis for the use of antifolates, which are used for cancer treatment via preventing de novo nucleotide biosynthesis due to the inhibition of folate enzymes [60]. Although the underlying mechanism has yet to be verified and confirmed, the observed phenomena and effect in the previous studies was attributed to DNA methylation reactions as a function of folate [58]. Therefore, the related gene-nutrition interaction may be far more complex than it originally seemed.

Despite decades of research on dietary or genetic factors which are related to cancer, resulting in a staggering number of publications, researchers are only beginning to grasp the complexity of the gene-nutrition interactions and pathways. Based on our results, one of the important implications of this study was to promote prevention of esophageal carcinogenesis by developing nutritional strategies that regulate epigenetics. However, owing to the limitations of case-control studies in establishing a causal relationship, low serum folate level might also be a consequence for ESCC. Patients are likely to have poor nutritional status due to cachexia; thus, tumor growth may cause a metabolic disorder of folate. Therefore, further large-scale prospective studies are needed to contribute to a deeper and more extensive understanding of genetics, epigenetics, nutrition, environmental factors, and their interactions in the different stages of carcinogenesis.

## Conclusion

Individually, both the folate level and the methylation status of p16 and p53 promoter regions may be associated with the risks of EPL and ESCC. However, they may interact in ways which has a different effect on the EPL and ESCC risks, suggesting the possible crosstalk between folate and aberrant DNA methylation. The present study evaluated the gene-nutrition interaction between folate and aberrant DNA methylation in the different stages of carcinogenesis of ESCC in a high risk rural area. A high serum folate level, high intake of folate-rich foods, and unmethylated p16 and p53 promoter regions may interact in ways which has a strong preventive effect on esophageal carcinogenesis. In addition, high serum folate level and high intake of foods rich in folate may offset the tumor-promoting effects of aberrant DNA methylation of p16 and p53 genes. Of note, a very high level of folate status may not have protective effect on EPL in some cases.

## Supplementary information

**Additional file 1: Figure S1**. Location of Huai'an District which is circled. Reprinted from OpenStreetMap, June 2 2020, retrieved from https://www.openstreetrnap.org/relation/5669085#map=9/33.5242/ 119.3225&layers=C. Copyright by OpenStreetMap Foundation contributors. Figure was edited with Microsoft Word 2007 (Microsoft Inc., Redmond, WA, USA) (https://www.microsoft.com/en-us/).

**Additional file 2: Table S1**. Folate content of common food (μg/100 g edible part).

**Additional file 3: Table S2**. Crude ORs (and 95% CIs) for dietary factors with EPL and ESCC (significant data).

**Additional file 4: Table S3**. Adjusted ORs (and 95% CIs) for dietary factors with EPL and ESCC (significant data).

## Data Availability

All data generated or analyzed during this study are included in the article and its supplementary information files.

## Abbreviations

ANOVA: Analysis of variance
CI: Confidence intervals
EC: Esophageal cancer
EDETPEC: Early Diagnosis and Early Treatment Project of Esophageal Cancer
ELISA: Enzyme-linked immunosorbent assay
EPL: Esophageal precancerous lesion
ESCC: Esophageal squamous cell carcinoma
FFQ: Food frequency questionnaire
MTHFR: Methylenetetrahydrofolate reductase
MSP: Methylation-specific polymerase chain reaction
OD: Optical density
OR: Odds ratios
