# Supplementary figures and images for: Moderately high folate level may offset the effects of aberrant DNA methylation of P16 and P53 genes in esophageal squamous cell carcinoma and precancerous lesions
Source: Genes Nutr. 2020 Sep 29;15:18. doi: 10.1186/s12263-020-00677-x (PMC7526188; doi:10.1186/s12263-020-00677-x)

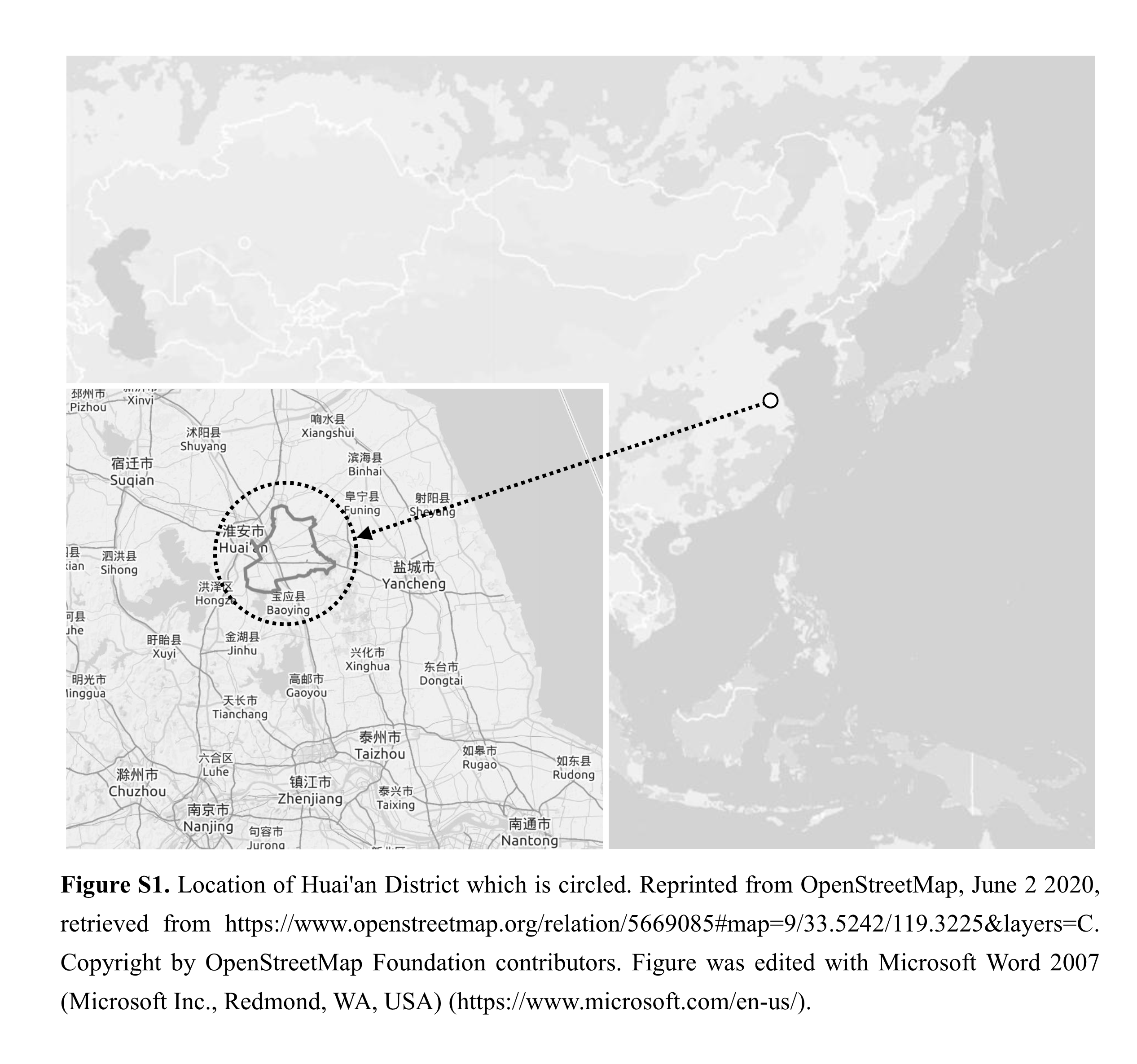

Supplement: Supplementary file 1 — Additional file 1: Figure S1. Location of Huai'an District which is circled. Reprinted from OpenStreetMap, June 2 2020, retrieved from https://www.openstreetrnap.org/relation/5669085#map=9/33.5242/ 119.3225&layers=C. Copyright by OpenStreetMap Foundation contributors. Figure was edited with Microsoft Word 2007 (Microsoft Inc., Redmond, WA, USA) (https://www.microsoft.com/en-us/). [file 12263_2020_677_MOESM1_ESM.png]
