# Supplementary material for: Moderately high folate level may offset the effects of aberrant DNA methylation of P16 and P53 genes in esophageal squamous cell carcinoma and precancerous lesions
Source: Genes Nutr. 2020 Sep 29;15:18. doi: 10.1186/s12263-020-00677-x (PMC7526188; doi:10.1186/s12263-020-00677-x)
Supplement: Supplementary file 2 — Additional file 2: Table S1. Folate content of common food (μg/100 g edible part). [file 12263_2020_677_MOESM2_ESM.docx]

**Table S1 Folate content of common food (μg/100 g edible part)**

| **Food item** | **Folate content** |  | **Food item** | **Folate content** |  | **Food item** | **Folate content** |
| --- | --- | --- | --- | --- | --- | --- | --- |
| Coriander | 148.8 |  | Citrus | 52.9 |  | Soybean | 181.1 |
| Crown daisy | 114.3 |  | Strawberry | 31.8 |  | Mung bean | 393.0 |
| Amaranthus tricolor | 419.8 |  | Pineapple | 25.0 |  | Adzuki bean | 87.9 |
| Spinach | 87.9 |  | Banana | 20.2 |  | Yuba | 48.4 |
| Garlic bolt | 90.9 |  | Hawthorn | 24.8 |  | Corn flour | 45.1 |
| Baby bok choy | 57.2 |  | Pork liver | 335.2 |  | Corn | 10.4 |
| Garlic chives | 61.2 |  | Sheep liver | 226.5 |  | Peanut | 107.5 |
| Chinese cabbage | 103.9 |  | Egg | 113.3 |  | Walnut | 102.6 |
| Lettuce | 22.7 |  | Chicken liver | 1172.2 |  | Dried mushroom | 110.0 |
| Tomato | 5.6 |  | Duck egg | 125.4 |  | Dried laver | 116.7 |
| Orange | 26.4 |  | - | - |  | - | - |
