# Supplementary material for: Moderately high folate level may offset the effects of aberrant DNA methylation of P16 and P53 genes in esophageal squamous cell carcinoma and precancerous lesions
Source: Genes Nutr. 2020 Sep 29;15:18. doi: 10.1186/s12263-020-00677-x (PMC7526188; doi:10.1186/s12263-020-00677-x)
Supplement: Supplementary file 3 — Additional file 3: Table S2. Crude ORs (and 95% CIs) for dietary factors with EPL and ESCC (significant data). [file 12263_2020_677_MOESM3_ESM.docx]

**Table S2 Crude ORs (and 95% CIs) for dietary factors with EPL and ESCC (significant data)**

| **Food variables** | **Crude OR (95% CI)** | ***p* value** |
| --- | --- | --- |
| Esophageal precancerous lesions | | |
| Spinach | 0.77 (0.60-0.99) | 0.040 |
| Chinese cabbage | 0.49 (0.36-0.66) | ˂0.001 |
| Citrus | 0.77 (0.60-0.98) | 0.033 |
| Strawberry | 0.73 (0.56-0.97) | 0.022 |
| Pineapple | 0.74 (0.56-0.97) | 0.027 |
| Egg | 0.65 (0.50-0.85) | 0.001 |
| Livers | 0.55 (0.36-0.83) | 0.004 |
| Beans | 0.78 (0.64-0.94) | 0.010 |
| Corn | 1.45 (1.08-1.94) | 0.014 |
| Peanut | 1.39 (1.06-1.83) | 0.019 |
| Esophageal squamous cell carcinoma | | |
| Amaranthustricolor | 0.68 (0.43-0.74) | 0.002 |
| Spinach | 0.46 (0.36-0.59) | ˂0.001 |
| Chinese cabbage | 0.72 (0.56-0.93) | 0.010 |
| Citrus | 0.63 (0.49-0.81) | ˂0.001 |
| Strawberry | 0.53 (0.39-0.71) | ˂0.001 |
| Pineapple | 0.51 (0.37-0.68) | ˂0.001 |
| Egg | 0.52 (0.40-0.67) | ˂0.001 |
| Livers | 0.58 (0.39-0.87) | 0.008 |
| Beans | 0.81 (0.67-0.98) | 0.031 |
| Corn | 1.61 (1.23-2.12) | 0.031 |
| Peanut | 0.69 (0.57-0.82) | 0.001 |
