# Supplementary material for: Moderately high folate level may offset the effects of aberrant DNA methylation of P16 and P53 genes in esophageal squamous cell carcinoma and precancerous lesions
Source: Genes Nutr. 2020 Sep 29;15:18. doi: 10.1186/s12263-020-00677-x (PMC7526188; doi:10.1186/s12263-020-00677-x)
Supplement: Supplementary file 4 — Additional file 4: Table S3. Adjusted ORs (and 95% CIs) for dietary factors with EPL and ESCC (significant data). [file 12263_2020_677_MOESM4_ESM.docx]

**Table S3 Adjusted ORs (and 95% CIs) for dietary factors with EPL and ESCC (significant data)**

| **Food variables** | **Adjusted OR (95% CI)*** | ***p* value** |
| --- | --- | --- |
| Esophageal precancerous lesions | | |
| Chinese cabbage | 0.47 (0.30-0.73) | 0.001 |
| Livers | 0.40 (0.22-0.71) | 0.002 |
| Beans | 0.46 (0.32-0.66) | ˂0.001 |
| Esophageal squamous cell carcinoma | | |
| Spinach | 0.61 (0.40-0.94) | 0.025 |
| Livers | 0.38 (0.20-0.71) | 0.003 |
| Beans | 0.66 (0.45-0.98) | 0.038 |

^*^ Adjusted for gender, age, tobacco smoking and alcoholic drinking.
